# Human Transbodies to Reverse Transcriptase Connection Subdomain of HIV-1 Gag-Pol Polyprotein Reduce Infectiousness of the Virus Progeny

**DOI:** 10.3390/vaccines9080893

**Published:** 2021-08-12

**Authors:** Watee Seesuay, Siratcha Phanthong, Jaslan Densumite, Kodchakorn Mahasongkram, Nitat Sookrung, Wanpen Chaicumpa

**Affiliations:** 1 Graduate Program in Immunology, Department of Immunology, Faculty of Medicine Siriraj Hospital, Mahidol University, Bangkok 10700, Thailand; watee.see@student.mahidol.ac.th (W.S.); jaslan.den@student.mahidol.ac.th (J.D.); 2 Center of Research Excellence on Therapeutic Proteins and Antibody Engineering, Department of Parasitology, Faculty of Medicine Siriraj Hospital, Mahidol University, Bangkok 10700, Thailand; kodchakorn.mah@mahidol.ac.th (K.M.); nitat.soo@mahidol.ac.th (N.S.); 3 Center of Excellent Research of Allergy and Immunology, Department of Research, Faculty of Medicine Siriraj Hospital, Mahidol University, Bangkok 10700, Thailand; siratcha.pha@mahidol.ac.th; 4 Biomedical Research Incubation Unit, Department of Research, Faculty of Medicine Siriraj Hospital, Mahidol University, Bangkok 10700, Thailand

**Keywords:** human single-chain antibodies (HuscFvs), cell-penetrating antibodies (transbodies), human immunodeficiency virus 1 (HIV-1), reverse transcriptase connection subdomain (RTCD), Gag-Pol polyprotein

## Abstract

HIV-1 progeny are released from infected cells as immature particles that are unable to infect new cells. Gag-Pol polyprotein dimerization via the reverse transcriptase connection domain (RTCDs) is pivotal for proper activation of the virus protease (PR protein) in an early event of the progeny virus maturation process. Thus, the RTCD is a potential therapeutic target for a broadly effective anti-HIV agent through impediment of virus maturation. In this study, human single-chain antibodies (HuscFvs) that bound to HIV-1 RTCD were generated using phage display technology. Computerized simulation guided the selection of the transformed *Escherichia coli*-derived HuscFvs that bound to the RTCD dimer interface. The selected HuscFvs were linked molecularly to human-derived-cell-penetrating peptide (CPP) to make them cell-penetrable (i.e., become transbodies). The CPP-HuscFvs/transbodies produced by a selected transformed *E. coli* clone were tested for anti-HIV-1 activity. CPP-HuscFvs of transformed *E. coli* clone 11 (CPP-HuscFv11) that presumptively bound at the RTCD dimer interface effectively reduced reverse transcriptase activity in the newly released virus progeny. Infectiousness of the progeny viruses obtained from CPP-HuscFv11-treated cells were reduced by a similar magnitude to those obtained from protease/reverse transcriptase inhibitor-treated cells, indicating anti-HIV-1 activity of the transbodies. The CPP-HuscFv11/transbodies to HIV-1 RTCD could be an alternative, anti-retroviral agent for long-term HIV-1 treatment.

## 1. Introduction

HIV is a retrovirus that belongs to the genus *Lentivirus* within the family Retroviridae. HIV infects vital cells of the human immune system, specifically monocytes/macrophages and CD4^+^ T cells, by using cell surface CD4 molecules as a primary receptor along with the human chemokine receptors, CCR5 and CXCR4, as co-receptors [1,2,3]. The infection also subverts dendritic cell functions to enhance the virus entry to key target cells and evade immune mechanisms for virus perpetuation and transmission [4]. Long-term HIV infection without treatment leads to progressive immune deficiency and fatal opportunistic infections and/or cancer, called acquired immunodeficiency syndrome (AIDS). HIV is classified into two types, HIV-1 and HIV-2. The great majority of infection globally is caused by HIV-1 [5]. HIV-1 was found to be more virulent and more infectious than HIV-2 [6]. However, unlike globally circulating strains, the CRF01_AE strain of HIV-1 is most prevalent in Thailand and neighboring countries in East and Southeast Asia [7]. Apparently, the CRF01_AE strain shows a stronger pathogenic virulence and is associated with faster AIDS progression [8,9]. Mutations of this strain in response to anti-retroviral therapy (ART) seem to occur relatively quickly compared to other virus subtypes [10]. Despite the success of anti-retroviral therapy (ART) by chemical drugs in significantly lessening the morbidity and mortality as a result of AIDS, long-term treatment with ART caused markedly severe toxic effects including bone, muscular, cardiovascular, neurological, liver, and immune disorders, as well as glucose and lipid metabolism disturbances, and death from non-AIDS causes [11]. There is also an emergence of ART-resistant/escape mutants [12]. Furthermore, the number of HIV-infected cells can persist and the latent reservoirs are unaffected by the current anti-retroviral agents [13,14]. Research on safe and effective therapeutic agents for HIV infection is still required.

Maturation of newly budded HIV-1 progeny is mediated by proteolytic cleavage of Gag and Gag-Pol polyproteins by the viral PR protein (protease), which is triggered by dimerization of Gag-Pol polyproteins (Gag-Pol) that were incorporated to the particles during virus assembly. The molecular interaction of Gag-Pol molecules occurs via reverse transcriptase (RT) domains, which was evident by the finding that Efavirenz, the first-generation non-nucleoside reverse transcriptase inhibitor (NNRTI), enhanced the RT dimerization [15] and accelerated proteolytic processing of the Gag and Gag-Pol polyproteins at the plasma membrane [16]. Resistance to Efavirenz-mediated enhancement of premature protease activation is observed in the virus mutants with mutation in the RT or RT tryptophan repeat motif [16,17]. Furthermore, alanine substitutions at tryptophan residues in the tryptophan repeat motif of RTCD impair PR activity of the released virions or causes premature activation of PR, which cleaves Gag and Gag-Pol polyproteins early in the cytosol [18]. The former causes a defect in the virus particle maturation resulting in non-infectious virus production, while the latter affects virion assembly leading to a marked reduction in number of properly assembled virus progeny. Moreover, mutations in the tryptophan residues in the RTCD of the Gag-Pol polyprotein impair the maturation and infectivity of the budded virions [18]. Thus, targeting the evolutionarily highly conserved epitopes of the RTCD in Gag-Pol polyprotein of HIV-1 could eventually restrict the progeny virus maturation and infectiousness. In this study, human single-chain antibodies (V_H_ and V_L_ domains linked via a peptide linker) in a cell-penetrable format (transbodies) that presumptively bind to RTCD at the dimer interface were generated. The transbodies markedly inhibited reverse transcriptase activity in the HIV-1 progeny particles, leading to a significant loss of the progeny virus infectivity.

## 2. Materials and Methods

### 2.1. Virus, Cells, Media, Plasmids, and Phage Display Library Used in This Study

Laboratory-adapted HIV-1_DA5_ (CXCR4 tropic CRF01_AE strain), originally isolated from HIV-infected Thai subjects [19], and H9 cell line (a clonal derivative of HuT78 which is a derived from human T lymphoblast cell) were obtained from the Department of Microbiology, Faculty of Medicine Siriraj Hospital, Mahidol University, Bangkok, Thailand. RPMI-1640 medium supplemented with 10% (*v*/*v*) heat-inactivated fetal bovine serum, 2.05 mM l-alanyl-l-glutamine, and ZellShield^®^ combined antibiotics (complete medium) was used to maintain H9- and HIV-1_DA5_-infected (H9^DA5^) cells. HuscFv phage display library constructed previously [20] was used.

*E. coli* NiCoR2 (DE3) and NiCoYC (DE3) were modified in-house from *E. coli* NiCo21 (DE3). They harbored pRARE2 and pYidC auxiliary plasmids, respectively. The plasmid pRARE2 was from *E. coli* Rosetta 2 (DE3), while the plasmid pYidC was constructed by cloning *yidC* (accession number NC_012971.2) into a pACYC vector backbone (Figure S1). The expression vector, pET24DAS, was constructed by replacement of the gene expression cassette of pET24a(+) at the sequence between *Nde*I and *Xho*I restriction sites with DNA encoding DsbA1 signal peptide (MKKIWLALAGLVLAFSASA), human Annexin III-derived-AA3H cell-penetrating peptide (ASIWVGHRG) [21], redesigned multiple cloning site, Strep-tag II epitope (WSHPQFEK), and tandem stop codons (Figure S2). Luria-Bertani (LB) broth was used for bacterial starter preparation and propagation, while the 2YT broth was used specifically to culture the phagemid-transformed *E. coli* HB2151 for soluble HuscFv expression. A basal medium for bacterial protein expression, ZYM-802, that contained 10 g/L Bacto™ tryptone, 5 g/L Bacto™ yeast extract, 25 mM Na_2_HPO_4_, 25 mM KH_2_PO_4_, 50 mM NH_4_Cl, 5 mM Na_2_SO_4_, 0.8% (*w*/*v*) glycerol, 0.02% (*w*/*v*) d-glucose, 0.005% (*v*/*v*) antifoam 204, and trace elements, was developed based on auto-induction medium [22] and optimized carbon source ratio [23].

### 2.2. HIV-1_DA5_ Genome Assembly

Genomic RNA of the HIV-1_DA5_ was isolated from cell-free culture medium containing the viral particles by using a Viral Nucleic Acid Extraction Kit II (Geneaid Biotech, Taiwan). Two hundred nanograms of purified viral RNA was used as a template for synthesis of first strand cDNA by RevertAid™ H Minus First Strand cDNA Synthesis Kit (Thermo Fisher Scientific, Waltham, MA, USA) using Anchored Oligo(dT)_20_ primer (Thermo Fisher Scientific) to prime cDNA synthesis at the 5′-poly(A) tail of the viral positive-sense RNA genome. The RNA template and the primer were allowed to react at 25 °C for 5 min before adding the other reaction mixture. First strand cDNA synthesis was performed at 42 °C for 1 h, followed by reaction termination at 70 °C for 5 min.

Five overlapping DNA segments of HIV-1_DA5_ genome were designed to encompass the entire viral genome including: R segment of 5′-LTR region to PR-coding segment of *pol* (*RU5-PR*, 2106 bp); PR- to IN-coding segment of *pol* (*PR-IN*, 2844 bp); IN-coding segment of *pol* to first exon of *tat* (*IN-NT*, 1415 bp); first to second exon of *tat* (*NT-CT*, 2613 bp); and second exon of *tat* to R segment of 3′-LTR region (*CT-RU3*, 1244 bp). Specific primers for all overlapped genome segments (Table S1) were designed from the closely related HIV-1 93TH902.1 isolate (accession number AF164485.1). Amplification of the DNA segments was performed by using Phusion™ Hot Start II High-Fidelity PCR Master Mix (Thermo Fisher Scientific) with 2 μL of the reverse transcription mixture containing the cDNA as a template. The amplified DNA fragments at the respective sizes were purified from agarose gel by using GenepHlow™ Gel/PCR Kit (Geneaid Biotech). The fragments were ligated into pJET1.2 cloning vector by using CloneJET™ PCR Cloning Kit (Thermo Fisher Scientific), and subsequently introduced to *E. coli* JM109 by using TransformAid™ Bacterial Transformation Kit (Thermo Fisher Scientific) according to the manufacturer’s instruction. The inserted DNAs were verified by using DNA Sequencing Services (Apical Scientific, Malaysia). Both forward and reverse sequencing reactions were performed by using pJET1.2-sequencing primers. For large DNA insert, primer-walking reactions were carried out by using internal sequencing primers, which were designed from the previous single sequence read, in order to determine the internal sequence. All sequences were then assembled de novo, generating a reference set of HIV-1_DA5_ genome assembly.

### 2.3. Production of Recombinant HIV-1 RTCD

Coding sequence of RTCD was retrieved from HIV-1_DA5_ reference genome assembly in order to design RTCD-specific primers. The primers were flanked at 5′-end with a ligation-independent cloning sequence extension, complementary to the cloning site of pLATE52 expression vector. DNA amplification was performed by using Phusion™ Hot Start II High-Fidelity PCR Master Mix (Thermo Fisher Scientific) with the recombinant plasmid containing the *PR-IN* segment as template. The amplified DNA amplicon at the expected size was purified and cloned directionally into the vector by using aLICator™ LIC Cloning Kit 4 (Thermo Fisher Scientific). The annealing mixture was introduced to *E. coli* JM109 competent cells. After sequence verification, recombinant RTCD expression plasmid (pLATE52-*RTCD*) was introduced to *E. coli* NiCoR2 (DE3) expression host and then the transformant was propagated in LB-ACG medium [LB broth supplementing with 100 μg/mL ampicillin, 34 μg/mL chloramphenicol, and 1% (*w*/*v*) d-glucose]. Recombinant RTCD expression was carried out by diluting the overnight bacterial culture in LB-ACG at 1:1000 in ZYM-802-40-AC (ZYM-802 supplementing 40 μM isopropyl β-d-1-thiogalactopyranoside, 100 μg/mL ampicillin, and 34 μg/mL chloramphenicol). The culture was grown at 24 °C with 220 rpm shaking for 24 h. Soluble recombinant RTCD with the N-terminal 6× His-tag was purified by immobilized metal affinity chromatography using TALON^®^ Metal Affinity Resin (Takara Bio Inc., Japan). Eluted fractions with high protein yields were pooled and dialyzed against non-buffered 8% (*w*/*v*) glycerol. Protein concentration was determined by using BCA protein assay. Purity of the recombinant protein was determined by using SDS-PAGE analysis.

### 2.4. In Vitro Production of HuscFvs That Bound to RTCD

Purified RTCD (200 nM concentration in 0.2 M carbonate-bicarbonate buffer, pH 9.4) was passively adsorbed on a microplate well surface. The unoccupied sites on the well surface were blocked by adding Protein-Free Blocking Buffer (Thermo Fisher Scientific) at 25 °C with 300 rpm-shaking for 1 h. The HuscFv phage display library was added to the well containing immobilized RTCD and kept at 25 °C with 300 rpm-shaking for 1 h. Unbound phages were removed by washing thoroughly with 0.1% (*w*/*v*) Tween-20 in phosphate-buffered saline, pH 7.4 (PBS). Phages that bound to the immobilized RTCD were rescued by incubating with mid-log phase grown *E. coli* HB2151. The phage-infected *E. coli* was spread on LB agar containing 100 μg/mL ampicillin and incubated at 37 °C overnight. Isolated colonies on the plate were cultivated individually in 150 μL of 2YT-AG medium (2YT broth supplemented with 100 μg/mL ampicillin and 2% (*w*/*v*) d-glucose) at 37 °C with 1000 rpm shaking for 1 h. Screening for HuscFv genes (*huscfvs*) was performed by PCR using *Taq* DNA Polymerase (Thermo Fisher Scientific), pCANTAB 5E-specific primers, and 1 μL of individual bacterial cultures as templates. The *E. coli* clones were selected based on the presence of an amplicon size of *huscfvs* at 1 kb. Expression of soluble HuscFvs from the *huscfv*-positive *E. coli* clones were performed by inoculating 1 μL of overnight culture of *E. coli* grown in 2YT-AG into 2 mL of fresh 2YT-A medium (2YT broth supplementing 100 μg/mL ampicillin) and grown at 30 °C with 250 rpm shaking for 16 h. Soluble protein fraction was extracted from the cells of each culture by using BugBuster^®^ Plus Benzonase^®^ Nuclease (Merck KGaA, Darmstadt, Germany) to lyse the cells at room temperature for 5 min. Cell debris and insoluble matter were removed by centrifugation at 15,000× *g*, 4 °C for 5 min. The supernatants were collected (soluble *E. coli* fractions).

Binding of HuscFvs in the soluble fractions of *E. coli* homogenates to RTCD was determined by using indirect ELISA. Wells of a microtiter plate were coated individually with 200 nM of RTCD (test antigen) or BSA (control antigen). Unoccupied sites on the well surface were blocked by using Protein-Free Blocking Buffer (Thermo Fisher Scientific) for 1 h. Individual soluble fractions of the *huscfv*-positive *E. coli* clones were diluted 1:2 with 0.05% (*w*/*v*) Tween-20 in PBS, pH 7.4 (PBS-T) before applying to the antigen-coated wells for 1 h and then the unbound proteins were washed away. Soluble protein fraction from *E. coli* host cells (HB) was included as background binding control. The wells were probed with rabbit anti-E tag antibody (Abcam, Cambridge, UK) (binds to an E-tag epitope linked at the HuscFv C-terminus). The RTCD-bound HuscFvs were then detected by adding goat anti-rabbit immunoglobulin (Ig)-HRP conjugate (SouthernBiotech, Birmingham, AL, USA). Each reaction step was kept at 25 °C with 300 rpm-shaking for 1 h. Washing with PBS-T between the steps was performed. Colorimetric signal was obtained by adding ABTS peroxidase substrate (Seracare Life Science, Milford, MA, USA). The optical density at 405 nm (OD 405 nm) was recorded at an appropriate time point against blank (a reaction without analyte and matrix). Positive binding was determined, i.e., OD 405 nm above mean + 3 standard deviations of HB (background binding). The cut-off value of 0.1 was used to exclude the clones for which their soluble extracts yielded a relatively low binding activity.

The presence of HuscFvs in the soluble extracts (positive binding) were confirmed by Western blot analysis. The proteins in soluble fractions of the *huscfv*-positive HB2151 *E. coli* homogenates were separated in the SDS-PAGE and then electro-blotted onto a nitrocellulose membrane. Unoccupied sites on the membrane were blocked with 5 % (*w*/*v*) skim milk in PBS. The membrane was probed with rabbit anti-E tag antibody (Abcam). Goat anti-rabbit Ig-AP conjugate (SouthernBiotech) was used for indirect detection of the HuscFv protein bands. Each step was kept at room temperature for 1 h. After washing away excess secondary antibody, the membrane was equilibrated with Tris-buffered saline (TBS), pH 9.5, at room temperature for 10 min before adding BCIP/NBT phosphatase substrate (Seracare Life Science). Once the bands on the membrane were clearly seen, the reaction was stopped by rinsing the membrane with deionized water.

### 2.5. In Silico Screening of RTCD-Bound HuscFvs

Individual phagemid of the *E. coli* clones that their products bound to RTCD were extracted. The *huscfv* inserts located between the 3′-end of *Sfi*I and 5′-end of *Not*I recognition sites were sequenced using pCANTAB 5E-specific primers as sequencing primers. V_H_- and V_L_-coding sequences in the inserts were identified by VBASE2 from Helmholtz Centre for Infection Research [24] (available at www.vbase2.org). The sequences were then deduced into polypeptide sequences and subjected to phylogenetic analysis by Phylogeny.fr from Information Génomique et Structurale [25] (available at www.phylogeny.fr). A representative of the sibling *E. coli* clones was selected for further analysis.

Identification of immunoglobulin framework regions (FRs) and complementarity-determining regions (CDRs) of the HuscFvs was performed by analysis of their variable fragment (Fv) models. Primary amino acid sequences of V_H_ and V_L_ domains were submitted to ABodyBuilder from Oxford Protein Informatics Group, University of Qxford [26] (available at opig.stats.ox.ac.uk) to generate the Fv structures. Martin’s antibody numbering scheme [27] was chosen to annotate the final models. North’s antibody CDR loop conformations [28] in the structure were clustered by submitting these models to PyIgClassify from Dunbrack Laboratory at Fox Chase Cancer Center [29] (available at dunbrack2.fccc.edu/PyIgClassify/). The CDR clusters were reported back as the primary sequences as well as the FR residues identifications. Non-hypervariable DE loops (CDR4) at FR3 of V_H_ [30] and V_L_ [31] were manually analyzed as these loops also formed CDR-like clusters.

Initial homologous models of RTCD and HuscFvs were generated by in silico prediction methods using I-TASSER from Yang Zhang’s Research Group, University of Michigan [32] (available at zhanglab.ccmb.med.umich.edu/I-TASSER/). For RTCD modeling, a polypeptide sequence deduced from RTCD expression construct was submitted to the I-TASSER. For HuscFvs modeling, both deduced HuscFv sequences and their respective Fv atomic coordinates were submitted to the I-TASSER. The Fv coordinate file with an alignment of the HuscFv sequence was used to specify the template structure and guided the I-TASSER to construct a full-length HuscFv atomic model. The homology models generated by the I-TASSER were chosen based on the highest C-score of the models. Each initial model was further refined to the near native state by ModRefiner [33] (available at zhanglab.ccmb.med.umich.edu/ModRefiner/). The refinement was performed in triplicate to generate three different conformational structures from each initial model. Then, high-resolution structures were obtained by submitting those refined models to FG-MD [34] (available at zhanglab.ccmb.med.umich.edu/FG-MD/). Thus, three conformational variant structures were obtained for individual RTCD and HuscFv homology models.

For molecular docking, all hydrogen atoms in the models were deleted from the structures. Virtual interactions between the modeled HuscFvs and RTCDs were predicted by using ClusPro 2.0 from Vajda Lab and ABC Group, Boston University and Stony Brook University [35] (available at cluspro.bu.edu). Antibody mode was manually selected, thus allowing asymmetric decoys as the reference state (ADARS) statistical potential [36] to drive the computational steps. The modeled HuscFv was submitted as a receptor and the modeled RTCD as a ligand. Exclusion of non-CDR regions from the docking simulation was carried out by manually uploading the FR masking file into a repulsion mode at the receptor column. The FR masking file was generated from the HuscFv model by deletion of all CDR and CDR-like clusters. Unstructured terminal residues of both the receptor and the ligand were removed by selection of a structure modification mode. The docking simulation was repeated nine times in a three-by-three block arrangement of the three conformational variant models of both RTCD and HuscFvs. All of the docking complexes in PDB file and model scores were retrieved from the server. Modeled RTCD–HuscFv complex was selected from a cluster with the highest number of docking members among the docking pairs. Binding interface of RTCD and HuscFvs from the representative model was analyzed by superimposing the retrieved model with a structure of HIV-1 RT (PDB ID: 4B3P). The RTCD in the model was structurally aligned with the RTCD structure (p66 of the RT). All modeled structures were visualized by using PyMOL 2.4.1 from Schrodinger (available at pymol.org). The HuscFvs that were positioned at the RTCD dimer interface were chosen.

### 2.6. Production of Cell-Penetrating HuscFvs (CPP-HuscFvs)

Coding sequences of HuscFvs that bound to the RTCD dimer interface were retrieved in order to design *huscfv*-specific primers. Forward and reverse primers were flanked at 5′-ends with *Nhe*I and *BsiW*I restriction sites, respectively. The DNA-coding HuscFv amplicons were ligated with pET24DAS by conventional ligation cloning using a Rapid DNA Ligation Kit (Thermo Fisher Scientific). The insert was sequenced to verify the presence of CPP-HuscFv-coding sequence by using pET24-specific primers. The expression vectors, pET24DAS-*huscfvs*, were introduced into *E. coli* NiCoYC (DE3) expression host and the transformant was propagated in LB-KCG medium (LB broth supplemented with 30 μg/mL kanamycin, 34 μg/mL chloramphenicol, and 1% (*w*/*v*) d-glucose).

Expression of CPP-HuscFvs was performed by diluting the overnight bacterial culture in LB-KCG at 1:1000 into ZYM-802-GSH-KC (ZYM-802 supplementing 5 mM reduced l-glutathione, 100 μg/mL kanamycin, and 34 μg/mL chloramphenicol). Each inoculum was then cultivated at 24 °C with 220 rpm shaking for 24 h allowing leaky expression of the CPP-HuscFvs. Turbidity of the culture before harvesting was determined at OD 600 nm. Cells were harvested from the liquid culture by centrifugation at 4000× *g*, 4 °C for 20 min, and stored at 4 °C until use.

Periplasmic protein extraction was initiated by resuspending the cell pellet with a cold TDRE buffer (200 mM Tris-HCl, pH 7.7 containing 0.025% (*w*/*v*) sodium deoxycholate, 50 mM l-arginine hydrochloride, and 50 mM l-glutamic acid sodium salt) at 1:10 of the original culture volume. To the cell suspension was added BioLock Biotin Blocking Solution (IBA GmbH, GÖttingen, Germany) to a final concentration of 0.2 units/mL, Benzonase^®^ Nuclease (Merck KGaA) to a final concentration of 2 units/mL, and MgCl_2_ to a final concentration of 2 mM. The mixture was kept stirring at 4 °C overnight in order to extract the periplasmic content. The preparation was centrifuged at 8000× *g*, 4 °C for 20 min; the supernatant was transferred to new container and NaCl and EDTA were added to final concentrations of 150 and 3 mM, respectively. The periplasmic extract was clarified by centrifugation at 12,000× *g*, 4 °C for 20 min before applying to affinity chromatography.

Soluble CPP-HuscFvs were purified from the periplasmic extract by StrepTrap XT Column (Cytiva, Marlborough, MA, USA) using ÄKTA start protein purification system (Cytiva). The column was equilibrated with TDRE buffer supplemented with 150 mM NaCl and 1 mM EDTA. The clear extract was loaded into the column at 1 mL/min flow rate. Column unbound contamination was removed by washing the column with WRE-100 buffer [WRE buffer containing 0.1% (*w*/*v*) Triton X-100]. Endotoxin contamination was removed by washing the column with cold WRE-114 buffer [WRE buffer containing 0.1% (*w*/*v*) Triton X-114]. Final wash was performed by using WRE buffer (20 mM HEPES buffer, pH 8.0 containing 50 mM l-arginine hydrochloride, 50 mM l-glutamic acid sodium salt, and 1 mM EDTA) until A_280_ value was stable. The captured CPP-HuscFvs were eluted from the column into several fractions by applying BRET buffer (20 mM HEPES buffer, pH 8.0 containing 50 mM D-biotin, 50 mM l-arginine hydrochloride, 50 mM l-glutamic acid sodium salt, and 60 mM trehalose). The eluted fractions that yielded high A_280_ value were pooled and diafiltrated by using Amicon^®^ Pro Purification System with 3K Ultra-0.5 Device (Merck KGaA). Biotin content was removed by exchanging with biotin-free BRET buffer. Protein concentration was determined by using Bradford protein assay. Purity of the recombinant CPP-HuscFvs was analyzed by using SDS-PAGE and CBB staining.

### 2.7. Half Maximal Effective Concentration (EC50) of the CPP-HuscFvs

EC50 of CPP-HuscFvs was measured by using indirect ELISA against immobilized RTCD. After blocking the RTCD-coated wells, the CPP-HuscFvs to be tested and the control CPP-HuscFv (6.4 μM to 0.1 nM) in PBS-T were added into the appropriate wells, and kept at 25 °C for 1 h. The fluids were discarded; the wells were washed with PBS-T and blocked with Biotin Blocking Buffer (IBA GmbH) at 25 °C for 10 min. The CPP-HuscFvs that complexed with immobilized RTCD in the wells were recognized by using Strep-Tactin^®^ HRP Conjugate (IBA GmbH). After keeping at 25 °C for 1 h, the wells were washed and colorimetric signal was obtained by adding ABTS substrate (Seracare Life Science). OD 405 nm was recorded against blank (a reaction without analyte and matrix) at appropriate time points by using microplate reader. Dose–response curve was constructed and the EC50 value was extrapolated from the curve.

### 2.8. Co-Immunoprecipitation (Co-IP)

Physical interaction of the CPP-HuscFvs with the RTCD was performed in Co-IP binding buffer (20 mM sodium phosphate buffer, pH 8.0, containing 150 mM NaCl, 1 mM EDTA, 8% (*w*/*v*) glycerol, 0.1% (*w*/*v*) Triton X-100, 100 μg/mL BSA, and biotin blocking agent) and then captured by using MagStrep type3 XT beads (IBA GmbH). Briefly, 1 mL of Co-IP binding buffer containing 0.5 μM of each CPP-HuscFvs and 0.2 μM of RTCD was kept at 25 °C with 500 rpm shaking for 1 h. The mixture was transferred to a tube containing 10 μL of 5% suspension of equilibrated magnetic beads, mixed, and kept on ice for 30 min with mixing every 10 min. Then, the tube was placed in a magnetic separator and the supernatant was removed. The beads were washed three times with 100 μL of Co-IP washing buffer (the binding buffer without BSA and biotin blocking reagent). The complexes that were captured on the beads were eluted by adding 20 μL of Co-IP elution buffer (20 mM sodium phosphate buffer, pH 8.0, containing 150 mM NaCl, 100 mM D-biotin, and 8% (*w*/*v*) glycerol) at room temperature for 10 min. The beads were removed by using a magnetic separator while the supernatant was transferred to a new tube.

Dot-blot ELISA was used to detect the presence of CPP-HuscFvs and RTCD in the preparation. Briefly, 3 μL of each supernatant sample was dropped onto a nitrocellulose membrane and air dried at room temperature. The membrane was soaked with ultrapure water and unoccupied surface was blocked with 5% (*w*/*v*) skim milk in TBS, pH 7.4 at room temperature for 1 h. The blots were probed with a 1:3000 dilution of StrepMAB-Classic (IBA GmbH) or anti-His Tag (BioLegend, San Diego, CA, USA), in order to detect Strep-tagged CPP-HuscFvs or His-tagged RTCD, respectively. Detection was performed by probing the membrane with a 1:5000 dilution of goat anti-mouse Ig-AP Conjugate (SouthernBiotech) as a secondary antibody. Each step was kept at room temperature for 1 h with continuous rocking. Washing between each step was performed by rinsing five times with 0.05% (*w*/*v*) Tween-20 in TBS, pH 7.4 at room temperature for 5 min/wash with continuous rocking. After washing, the membranes were equilibrated with TBS, pH 9.5 at room temperature for 10 min. Color on the membranes was developed by adding BCIP/NBT phosphatase substrate (Seracare Life Science) at room temperature until the dots were clearly visible. The reaction was stopped by rinsing the membrane with deionized water. The color intensities of the dots were quantified by using ImageJ from National Institutes of Health (available at imagej.nih.gov/ii/).

### 2.9. Preparation of HIV-1-Infected Cells

HIV-1_DA5_ (containing 1 × 10^9^ copies) was added to the 1 × 10^6^ H9 cells maintained in complete medium and incubated in CO_2_ incubator at 37 °C for 24 h. The cells were then washed with Hank’s Balanced Salt Solution (HBSS) containing Calcium/Magnesium (Thermo Fisher Scientific) by centrifugation, replenished with fresh complete medium and incubated further. Virus particles in spent culture medium were determined at 24 h-intervals for 15 days by using HIV Ag/Ab Test Kit (Fujirebio, Tokyo, Japan). The infected cells collected at day 15 of infection were cryopreserved in small portions. The percentage of virus-producing cells was checked by measuring intracellular HIV-1 p24 from 1 × 10^6^ infected cell aliquots.

### 2.10. Biocompatibility of CPP-HuscFvs

Biocompatibility of the CPP-HuscFvs to H9 and H9^DA5^ cells was determined by using CellTiter-Glo^®^ Luminescent Cell Viability Assay (Promega Corp., Madison, WI, USA) in which the ATP quantity in the cell culture correlates with the cell viability. Briefly, 50 μL of complete medium containing 2, 20, 200 nM, and 2 μM of CPP-HuscFvs were added into 50 μL of the medium containing 5 × 10^4^ H9 or H9^DA5^ cells in wells of a white 96-well assay plate (Corning Inc., Corning, AZ, USA) and incubated at 37 °C in CO_2_ incubator for 24 h. Individual treatments including controls were performed in triplicate. After incubation, the cultures were equilibrated at room temperature for 30 min. The cells in each well were lysed by adding 100 μL of the CellTiter-Glo Reagent and mixed well for 2 min. Then, the plate was kept at room temperature in darkness for 10 min for luminescence stabilization before recording the signals.

### 2.11. Cell-Penetrating Ability and Cellular Localization of CPP-HuscFvs

H9^DA5^ (2 × 10^5^ cells in 200 μL complete medium containing 15 μg/mL of CPP-HuscFvs) was placed into wells of a 48-well Cell Culture Plate (Eppendorf AG, Germany) and incubated at 37 °C in CO_2_ incubator for 6 h. The cells were harvested by centrifugation at 10,000× *g*, 4 °C for 1 min. The cell pellet was resuspended with 200 μL of ice-cold Dulbecco’s PBS (DPBS) without Calcium/Magnesium (Thermo Fisher Scientific), fixed by adding 200 μL of eBioscience™ IC Fixation Buffer (Thermo Fisher Scientific), and kept at room temperature for 20 min. The fixative was removed by centrifugation at 10,000× *g*, 4 °C for 1 min, and the cells were rinsed with 1 mL of eBioscience™ Flow Cytometry Staining Buffer (Thermo Fisher Scientific). Cell permeabilization was performed by adding 200 μL of eBioscience™ Permeabilization Buffer (Thermo Fisher Scientific). The permeabilized cells were stained for intracellular CPP-HuscFvs and Gag-Pol polyprotein. The cells were incubated with 1:100 dilution of StrepMAB-Classic (IBA GmbH) followed by staining with 1:300 dilution of goat anti-mouse IgG-Alexa Fluor^®^ 555 conjugate (Thermo Fisher Scientific). For intracellular Gag-Pol polyprotein staining, the cells were incubated with 1:200 dilution of rabbit anti-HIV-1 reverse transcriptase antibody (Abcam) followed by incubation with 1:500 dilution of goat anti-rabbit IgG-Alexa Fluor^®^ 488 Conjugate (Abcam). Nuclei were located by staining with 1 μg/mL Hoechst 33342 (Biotium Inc., Fremont, CA USA). The cells were mounted on a glass slide by using EverBrite™ Hardset Mounting Medium (Biotium) and analyzed by confocal microscopy.

### 2.12. Treatment of HIV-1 Infected Cells

HIV-1 infected cells maintained in complete medium were washed twice with HBSS containing Calcium/Magnesium (Thermo Fisher Scientific) and resuspended at 1 × 10^6^ cells/mL in the fresh complete medium. The cells were chilled on ice for 1 h before use. One hundred microliters of the medium containing 230 nM of CPP-HuscFvs, 10 nM of protease inhibitor, Darunavir, or 10 μM of nucleoside reverse transcriptase inhibitor, Tenofovir, were placed into appropriate wells of a 96-well Cell Culture Plate (Eppendorf AG). To each well was then added 100 μL of the infected cells and the plate was incubated at 37 °C in a CO_2_ incubator for 24 h. The cells were removed from the virus-containing culture medium by centrifugation at 250× *g*, 4 °C for 10 min. The supernatant (180 μL) was transferred to new microcentrifuge tube and then re-centrifuged at 8000× *g*, 4 °C for 10 min to remove cell debris. The clear supernatant was used subsequently in three different experiments including: HIV-1 viral load assay (5 μL of the supernatant were diluted 200-fold with DPBS without Calcium/Magnesium (Thermo Fisher Scientific); HIV-1 RT enzymatic activity assay (60 μL of the supernatant); and HIV-1 virus infection assay (50 μL of the supernatant). All portions of the supernatant were kept at −80 °C until use.

### 2.13. HIV-1 Viral Load Assay

Amount of HIV-1 p24 in the infected cells culture supernatant was determined by using Genscreen™ Ultra HIV Ag-Ab kit (Bio-Rad Laboratories, Hercules, CA, USA), according to the manufacturer’s instructions. Briefly, 75 μL of the 1:200 culture supernatant (from Section 2.12) were mixed with 25 μL of biotinylated sheep polyclonal antibodies to HIV-1 p24 in wells of a microplate provided with the kit, which was pre-coated with mouse monoclonal antibody to HIV-1 p24. The plate was kept at 25 °C with 300 rpm shaking for 1 min and then incubated at 37 °C without shaking for 1 h. The captured antigen–antibody complexes were detected by adding streptavidin–HRP conjugate at 25 °C for 30 min. Colorimetric signal was obtained by adding TMB peroxidase substrate and kept in darkness at 25 °C for 30 min. The reaction was stopped by adding 1 N H_2_SO_4_ and kept further in darkness at room temperature for 5 min. The intensities of the color were determined by measuring at OD 450 nm with a reference wavelength at OD 630 nm. The HIV-1 titer of each sample was determined from a standard curve constructed by plotting a linear regression of the blank-corrected OD 450 nm/630 nm for each known virus dilution against its titer (2^−N^).

### 2.14. HIV-1 RT Enzymatic Activity Assay

HIV-1 RT activity in the HIV-1 particles was measured by using Roche^®^ Colorimetric Reverse Transcriptase Assay kit (Merck KGaA). HIV-1 particles were isolated from the 60 μL culture supernatant (from Section 2.12) by using Lenti-X™ Concentrator (Takara Bio Inc.). The clarified supernatant was mixed with 20 μL of the concentrator reagent and kept at 4 °C overnight. The virus particles were pelleted by centrifugation at 1500× *g*, 4 °C for 45 min and 65 μL of the supernatant was removed. The remaining was re-centrifuged for 15 min and the supernatant was discarded. The virus pellet was resuspended in 40 μL of lysis buffer (provided with the kit) and kept at 20 °C for 30 min. The virus lysate (8 μL) was diluted 5-fold by adding 32 μL of the lysis buffer and mixed with 20 μL of provided reaction mixture containing poly(A)×oligo(dT)15. The reverse transcriptase reaction was carried out at 37 °C for 15 h. The reaction mixture (60 μL) was transferred to a well of a microplate module (provided with the kit), which was pre-coated with streptavidin and blocking reagent, and incubated at 37 °C for 1 h. Complementary DNA that was generated from the viral RNA was detected by adding anti-DIG-POD (provided with the kit) to each well, and the plate was incubated at 37 °C for 1 h. Colorimetric reaction was developed by adding ABTS substrate to each well and kept at 20 °C in darkness for 30 min. The intensities of the color were determined by measuring at OD 405 nm with a reference wavelength at OD 450 nm. RT activity of the virus sample was extrapolated from the RT activity standard curve constructed by plotting a linear regression of the blank-corrected OD 405/450 nm for each known virus lysate dilution against its titer (2^−N^).

### 2.15. HIV-1 Virus Infection Assay

On day 0, wells of a 48-well Cell Culture Plate (Eppendorf AG) were added with 150 μL of the complete medium containing 1 × 10^5^ H9 cells. The cells were then added 50 μL of the culture supernatant containing HIV-1 particles derived from the treatment experiment (Section 2.12). The contents (200 μL) were mixed by pipetting and incubated at 37 °C in a CO_2_ incubator for 48 h. On day 2, subculture was performed by transferring 150 μL of the infected culture into 450 μL of fresh complete medium that was placed in the well of a 24-well Cell Culture Plate. The contents (600 μL) were mixed and incubated further for another 48 h. On day 4, cells were harvested from 400 μL of each culture for intracellular HIV-1 p24 staining. Briefly, cells were sedimented and resuspended in 200 μL of ice-cold DPBS without Calcium/Magnesium (Thermo Fisher Scientific). They were fixed by adding 200 μL of eBioscience™ IC Fixation Buffer (Thermo Fisher Scientific) and kept at room temperature for 20 min. After removing the fixative agent, permeabilization was performed by adding 200 μL of eBioscience™ Permeabilization Buffer (Thermo Fisher Scientific). The suspension was briefly mixed and kept at room temperature for another 20 min. After removing the permeabilizing agent, the cells were resuspended in 195 μL of the permeabilization buffer containing 1:200 dilution of RedDot™ 1 Far-Red Nuclear Stain (Biotium). HIV-1 p24 in cytoplasm was stained by adding 5 μL of Coulter Clone KC57-FITC (Beckman Coulter Inc., Brea, CA, USA) and kept at room temperature with light protection for 20 min. The excessed antibody and dye were removed by centrifugation at 500× *g*, 4 °C for 5 min. The cells were then resuspended in 1 mL of eBioscience™ Flow Cytometry Staining Buffer (Thermo Fisher Scientific). In flow cytometry analysis, cells were determined by gating from FSC/SSC and RedDot staining. FITC fluorescence intensity of the cells was acquired with the gating being selected based on uninfected cells as reference. FITC and RedDot double-positive cells were considered as virus-producing cells. The flow cytometric data were analyzed and presented by using FlowJo 10.7 from BD Life Sciences (available at www.flowjo.com/solutions/flowjo).

### 2.16. Statistical Analysis

Each experiment was performed at least in triplicated. Prism 9 from GraphPad Software (available at graphpad.com/scientific-software/prism/) was used to compare the results (mean ± standard deviation (SD)) among different treatment groups. A *p*-value < 0.05 was taken as statistical significance.

## 3. Results

### 3.1. HIV-1_DA5_ Genome

**Figure 1:**
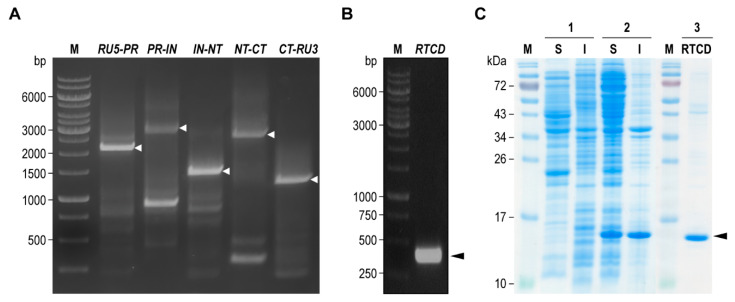
Amplicons of HIV-1_DA5_ genome fragments and recombinant RTCD preparation. (**A**) Amplicons of five genome fragments for HIV-1_DA5_ for whole genome de novo sequence assembly. Lane M, 1 kb DNA ladder in base pairs (bp). White arrowheads indicate the respective DNA amplicons. (**B**) Amplicon of RTCD-coding sequence (RTCD; 342 bp; black arrowhead) amplified from plasmids that contained *PR-IN* segment by using RTCD-specific primers. Lane M, 1 kb DNA ladder in bp. (**C**) Recombinant RTCD expressed in the transformed *E. coli* homogenate (column 2) compared to proteins in *E. coli* host homogenate (column 1). The RTCD (~16 kDa; black arrowhead) was purified from *E. coli* soluble fraction (column 3). Lanes M, pre-stained protein ladders; lanes S and I, soluble and insoluble fractions of the *E. coli* homogenates, respectively. Numbers at the left of (**C**) are protein masses in kDa.

Reference genome sequence of HIV-1_DA5_ was established through de novo assembly of the virus genomic fragments. Five overlapping DNA segments of HIV-1_DA5_ genome that encompassed the entire viral genome, including *RU5-PR* (2106 bp), *PR-IN* (2844 bp), *IN-NT* (1415 bp), *NT-CT* (2613 bp), and *CT-RU3* (1244 bp), were amplified from the cDNA template (Figure 1A). They were assembled by aligning the respective overlapped complementary segments of the sequences. The assembled HIV-1_DA5_ genome was organized based on HIV-1 93TH902.1 isolate as reference (Figure S3). The genome consists of 9678 nucleotides, which most of the genes encoding the HIV-1 proteins are functional, except *vpr* and *vpu*. The Vpr-coding gene has a frameshift mutation caused by a single nucleotide deletion at position 5639 of the genome. The Vpu-coding gene has a nonsense mutation caused by a single base substitution of guanine to adenine at position 6131. The mutations led to expression of nonfunctional Vpr and truncated Vpu, respectively.

### 3.2. Recombinant RTCD

The sequence encoding RTCD (*RT* 949-1257) was amplified from PR-IN genome fragment (Figure 1B). The DNA fragment was cloned into pLATE52 expression vector via ligation-independent cloning; thus, fused with 6× His and T7 tags at N-terminus (Figure S4). Soluble expression of recombinant RTCD was achieved by using IPTG-based auto-induction medium in the presence of pRARE2 auxiliary plasmid. Half of the expressed recombinant protein was found in a soluble (S) form (Lane 2, Figure 1C). The soluble RTCD was then purified by using metal affinity chromatography under native condition. The size of the recombinant RTCD determined by SDS-PAGE and CBB staining was approximately 16 kDa (Lane 3, Figure 1C; black arrowhead).

### 3.3. Binding of HuscFvs to RTCD

**Figure 2:**
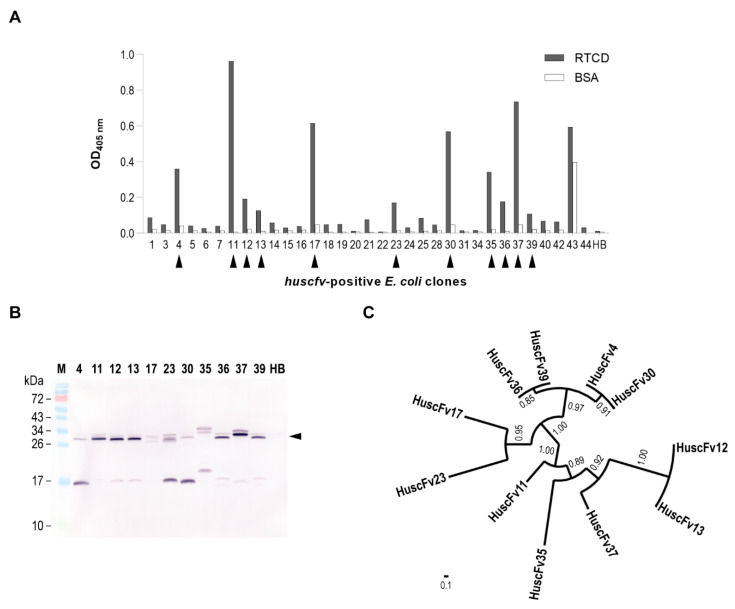
RTCD-bound HuscFvs. (**A**) Binding of HuscFvs in soluble fractions of 33 *huscfv*-positive *E. coli* HB2151 clones to immobilized RTCD by indirect ELISA. BSA was used as control antigen and HB, a soluble fraction of *E. coli* HB2151 host cell homogenate, served as negative (background) binding control. Black arrowheads indicate the *E. coli* clones that bound to RTCD and gave significant ELISA signals (OD 405 nm) above cut-off (i.e., OD 405 nm > 0.1) and at least twice the OD of the same preparation to BSA control. (**B**) Appearance of HuscFvs in soluble fractions of the *E. coli* clones that bound to RTCD. The HuscFvs are reactive bands at about 26–34 kDa (black arrowhead). The lower reactive bands indicated truncated fragments of the principal proteins. Lane M, pre-stained protein ladder; lane HB, soluble fraction of *E. coli* HB2151 host cell lysate. Numbers at the left are protein masses in kDa. (**C**) Phylogenetic analysis of HuscFvs of the 11 *E. coli* clones that bound to RTCD. The figure shows that some clones are sibling; thus, only 8 groups of HuscFvs could be obtained, i.e., group 1 (HuscFv4 and HuscFv30), group 2 (HuscFv11), group 3 (HuscFv12 and HuscFv13), group 4 (HuscFv17), group 5 (HuscFv23), group 6 (HuscFv35), group 7 (HuscFv36 and HuscFv39), and group 8 (HuscFv37).

Selection of RTCD-bound phages was performed in a bio-panning process. After intensive washing to remove unbound phages from the RTCD-immobilized well, the antigen-bound phages were incubated with log-phase *E. coli* HB2151 to allow recombinant phagemid transduction. The clones that were grown on selective agar were screened for the presence of *huscfvs* by using direct PCR. From 44 clones, 33 clones carried inserted *huscfvs*, as shown by the PCR amplicons at approximately 1 kb (Figure S5). The PCR-positive clones were cultured overnight in glucose-deprived selective broth, which allowed leaky expression of recombinant HuscFvs from *huscfv*-pCANTAB 5E. Soluble protein fractions from individual *E. coli* HB2151 homogenates were tested for binding to recombinant RTCD by indirect ELISA, and HuscFvs of 11 of the 33 *huscfv*-positive clones bound to immobilized RTCD (Figure 2A). The presence of HuscFvs in the lysates of these clones were confirmed by using Western blot analysis (Figure 2B). The *huscfvs* of the 11 clones were subjected to DNA sequencing and the sequences were deduced. The clones could be classified based on their amino acid sequences into eight groups, as shown in a circular phylogenetic tree (Figure 2C). They were group 1 (clones HuscFv4 and HuscFv30), group 2 (clone HuscFv11), group 3 (clones HuscFv12 and HuscFv13), group 4 (HuscFv17), group 5 (HuscFv23), group 6 (HuscFv35), group 7 (clones HuscFv36 and HuscFv39), and group 8 (clone HuscFv37). The clones 11, 12, 17, 23, 30, 35, 36, and 37 were chosen for in silico binding analysis of their HuscFvs to RTCD.

### 3.4. Computerized Simulation for Screening of HuscFvs

Molecular docking was used to predict the preferred orientation of HuscFvs from individual *E. coli* clones that formed complex with RTCD. Models of variable fragments (Fv) of individual HuscFvs that predicted from ABodyBuilder were submitted to I-TASSER for single-chain Fv modeling and to PyIgClassify for identification of structural-based CDRs. North’s antibody CDR loop clusters of both V_H_ and V_L_ domains were obtained and non-hypervariable DE loops at FR3 of V_H_ and V_L_ were manually identified. Information of the structural CDR clusters was used to generate framework region (FR) models.

Models of HuscFvs and RTCD with the highest c-score from I-TASSER (Table S2) were refined by using ModRefiner. Structural refinement was performed in triplicate to generate three energy-minimized models of individual HuscFvs and RTCD. The model (M1, M2, and M3) that were generated from a single model template showed notable differences in protein backbone conformation (Table S3). High-resolution structures were obtained from FG-MD by using the refined models as templates; hence generated rotameric state of side-chain conformations (F1, F2, and F3) of the HuscFv and RTCD models (Figure S6). Individual models of FRs were generated by deleting all CDR clusters from the obtained high-resolution HuscFv models (data not shown).

**Figure 3:**
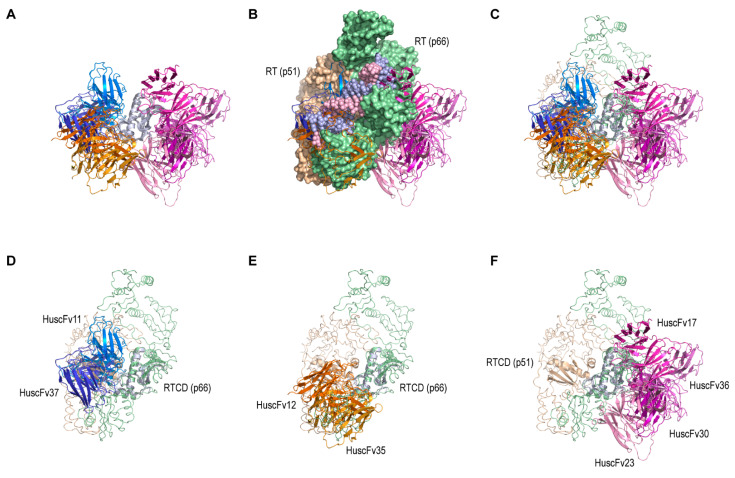
In silico screening of RTCD-bound HuscFvs. (**A**) Docking complexes of all HuscFvs with the modeled RTCD (grey). (**B**,**C**) Superimposition of connection domain in p66 (green surface and ribbon) of RT structure (4B3P) by the HuscFv-complexed RTCDs. RT p51 domain was indicated by beige surface and ribbon. (**D**) Binding of HuscFv11 and HuscFv37 (dark and light blue shades) to RTCD (p66). (**E**) Binding of HuscFv12 and HuscFv35 (orange shade) to RTCD (p66). (**F**) Binding of HuscFv17, HuscFv23, HuscFv30, and HuscFv36 (magenta shade) to the opposite site of RTCD (p51).

Modeled HuscFvs were subjected to computationally screening by intermolecular docking with the modeled RTCD in order to bin the epitopes of HuscFvs and for identification of the potential HuscFvs to be used in the subsequent bioassays. Molecular docking was performed in a three-by-three block arrangement (a total of nine docking pairs) and thus generated up to 270 docking clusters from individual HuscFvs. The cluster which contained the highest number of members, and claimed to be the most stable complex, was chosen for analysis of the binding interface between the HuscFv and the RTCD (Figure 3A and Table S4). The complex models were superimposed to RTCD of p66 of the RT (PDB ID: 4B3P) (Figure 3B,C). Thus, the binding positions could be divided into three different binning. The first binning included the HuscFvs that bound to the RTCD dimer interface and clashed with the RTCD of p51, which were formed by HuscFv11 and HuscFv37 (Figure 3D). The second binning included the HuscFv12 and HuscFv35 that bound with the RTCD portion that seemed to be protected by the RNase H domain (Figure 3E). The third binning was the HuscFv17, HuscFv23, HuscFv30, and HuscFv36 that bound to the β-barrel motif of RTCD opposite to the RTCD dimer interface (Figure 3F). Therefore, the HuscFv11 and HuscFv37 that clashed with the RTCD dimer interface were chosen for further bioassays.

### 3.5. Preparation of HIV-1_DA5_-Infected H9 Cells

**Figure 4:**
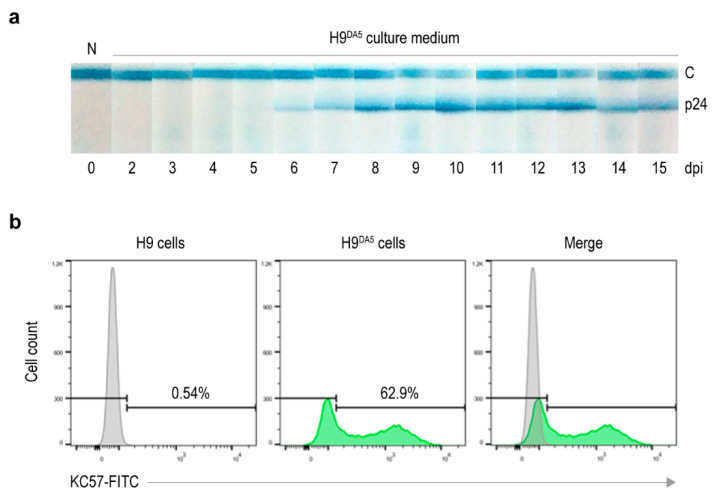
HIV-infected H9 (H9^DA5^) cells. (**a**) Kinetics of HIV-1_DA5_ production from H9^DA5^ cells. Amounts of p24 antigen in the culture supernatants of the HIV-1_DA5_-infected H9 cells were measured daily up to 15 days post infection (dpi). N, H9 culture medium alone; C, control line. (**b**) Histograms of H9^DA5^ cells at 15 dpi. Virus-producing cell population (62.9%) was determined by cells that positive for intracellular p24 staining (green). H9 cells were used as negative intracellular p24 staining.

Laboratory-adapted HIV-1_DA5_-infected H9 cells (H9^DA5^) were prepared by culturing H9 cells in the complete medium containing HIV-1_DA5_ particles for 15 days. The culture medium was monitored daily for virus particles. The virus particles started to appear on day 6 post infection and the peaked between days 8 and 13. The virus levels dropped on days 14 and 15 (Figure 4a). Thus, the H9^DA5^ cells were harvested on day 15 and cryopreserved. The percentage of virus-producing cell population at day 15 post infection was 62.9% (Figure 4b).

### 3.6. Characteristics of CPP-HuscFvs

**Figure 5:**
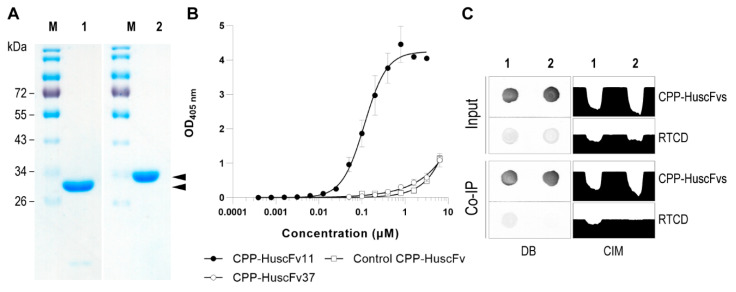
Recombinant CPP-HuscFv preparations and their binding activities. (**A**) Affinity-purified CPP-HuscFv11 (lane 1, lower arrowhead) and CPP-HuscFv37 (lane 2, upper arrowhead). Lanes M, pre-stained protein ladder. Numbers at the left are protein masses in kDa. (**B**) RTCD binding curve of the purified CPP-HuscFv11 (black circles), CPP-HuscFv37 (white circles), and control CPP-HuscFv (white squares). (**C**) Co-immunoprecipitation to demonstrate physiological binding of CPP-HuscFv11 (lane 1) and CPP-HuscFv37 (lane 2) to the RTCD in ELISA. DB, dot-blot ELISA; CIM, color intensity map.

DNA-coding HuscFv11 and HuscFv37 were subcloned into pET24DAS expression vector by using conventional ligation-dependent cloning. Production of the CPP-HuscFvs were achieved by leaking expression of the gene under T7 promoter and concurrently translocated into bacterial periplasmic space where DsbA1 signal peptide is cleaved off (co-translation-translocation). The periplasmic proteins were extracted from *E. coli* NiCoYC (DE3) host and the CPP-HuscFvs were purified under native condition. The purified antibody fractions were pooled and concentrated. Biotin content in the elution buffer was removed by diafiltration and the CPP-HuscFvs were characterized by SDS-PAGE and CBB staining (Figure 5A).

Effective concentration (EC) and binding activity of the CPP-HuscFvs to RTCD and control CPP-HuscFv were evaluated by using indirect ELISA and co-immunoprecipitation (Co-IP). The binding curve of CPP-HuscFv11 was saturated at 0.4 μM and its EC50 was 0.115 μM (3.28 μg/mL) (Figure 5B). However, the binding curve of CPP-HuscFv37 and control CPP-HuscFv did not show any saturated concentration even at 6.4 μM; thus, the EC50 of the CPP-HuscFv37 could not be calculated from the curve (Figure 5B). The CPP-HuscFv11 was also co-immunoprecipitated with RTCD while the interaction between CPP-HuscFv37 and RTCD could not be detected (Figure 5C). Therefore, only the CPP-HuscFv11 was tested further.

**Figure 6:**
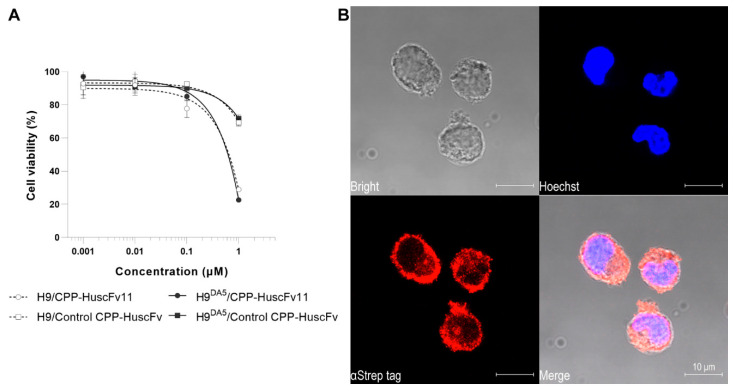
Biocompatibility and cell-penetrating ability of CPP-HuscFv11 to human cells. (**A**) Cell viability of H9/H9^DA5^ cells after treated with CPP-HuscFv11 or control CPP-HuscFv at varying concentrations (0.001, 0.01, 0.1, and 1 μM) for 24 h. The treatments were performed with an equivalent volume of antibody diluent. The relative cell viability was calculated by dividing by that of the medium control. (**B**) Cell-penetrating ability of CPP-HuscFv11 was examined by confocal microscopy. Cells were incubated with 15 μg/mL of CPP-HuscFv11 for 6 h. Intracellular localization of the CPP-HuscFv11 (transbodies) appeared in red color matter within the cells.

Biocompatibility of CPP-HuscFv11 to human cells was tested by using H9 and H9^DA5^ cells as representatives. Cells were treated for 24 h with 1 nM to 1 μM of the CPP-HuscFv11 and the cell viability was analyzed by measuring total ATP content in the culture. The CPP-HuscFv11 did not significantly affect the viability of the cells at concentrations up to 100 nM. Viability of the cells was markedly reduced when the CPP-HuscFv11 concentration was 1 μM (Figure 6A).

**Figure 7:**
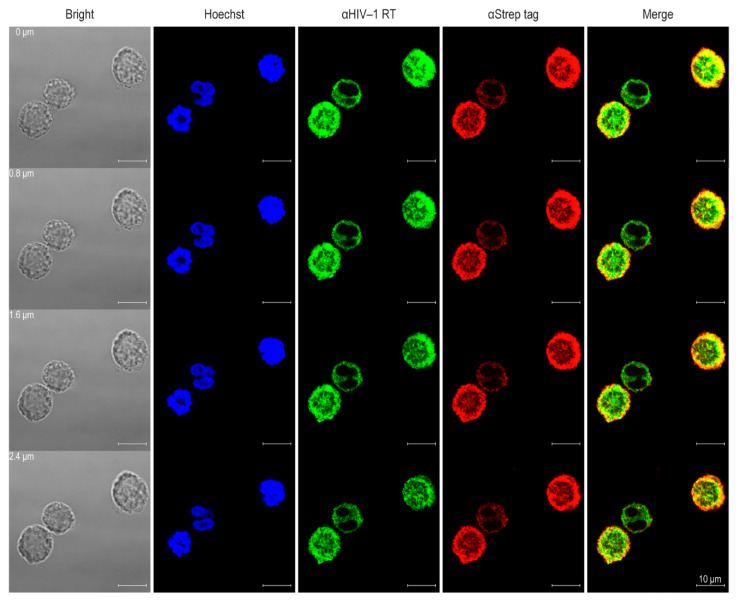
Co-localization of CPP-HuscFv11 (transbodies) to intracellular Gag-Pol polyprotein. H9^DA5^ cells treated with CPP-HuscFv11 for 6 h were analyzed by confocal microscopy. Left to right columns: bright field, nuclei stained blue by Hoechst; Gag-Pol labeled green by anti-HIV-1 RT; CPP-HuscFv11/transbodies labeled red by anti-Strep tag; and merge field (green and red), respectively. Co-localization of Gag-Pol and CPP-HuscFv11/transbodies was visualized (yellow/orange in merge). Different panels in the same column demonstrate the target entities in 0.8 μm laser sections from a series of z-stack analysis.

Cell internalization of CPP-HuscFv11 was demonstrated by confocal microscopy. H9^DA5^ cells were incubated for 6 h with CPP-HuscFv11. Intracellular retention of stained CPP-HuscFv11 and the fluorescence signal revealed that the antibody was able to translocate and was retained intracellularly (Figure 6B). Moreover, CPP-HuscFv11 was found to be co-localizing with intracellular Gag-Pol polyprotein in the infected cells (Figure 7).

### 3.7. Effects of CPP-HuscFvs/Transbodies to RTCD on HIV-1 Produced from Infected Cells

**Figure 8:**
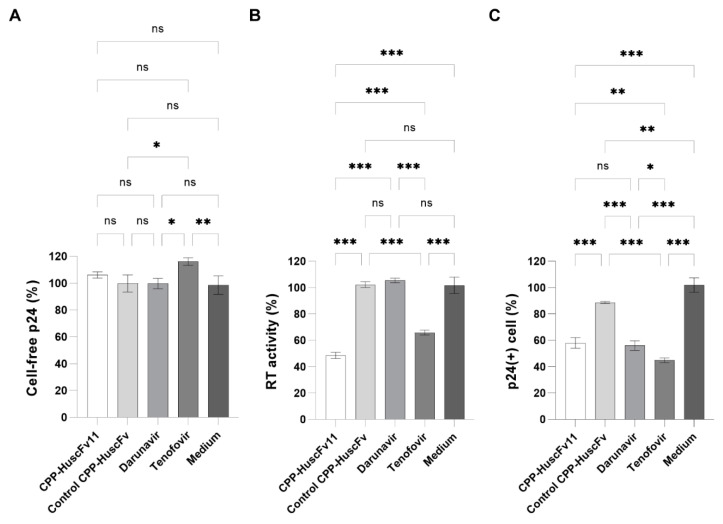
Viral load in culture medium, reverse transcriptase activity in the particles, and infectivity of the virus derived from HIV-infected cells treated with CPP-HuscFv11 and controls. Infected cells were treated with CPP-HuscFv11, control CPP-HuscFv, Darunavir, Tenofovir, and without any treatment (Medium). (**A**) Percent p24 in the cell-free culture medium containing the virus progeny of infected cells treated with CPP-HuscFv11 compared to Medium and other treatment controls. (**B**) Virus particles in each culture medium were isolated and the RT enzymatic activity was measured. (**C**) The virus progeny derived from the treated cells was used to determine the infectivity by employing virus infection assay. Result in (**A**–**C**) are shown as mean ± standard error of triplicates. Statistically significant differences were determined using one-way ANOVA with Dunnett multiple comparison test (95% confidence interval). ***, *p* < 0.001; **, 0.001 < *p* < 0.002; *, 0.002 < *p* < 0.05; ns, *p* ≥ 0.05 (not significantly different).

H9^DA5^ cells were treated for 24 h with CPP-HuscFv11/transbodies. Controls included control CPP-HuscFv, Darunavir protease inhibitor and Tenofovir reverse transcriptase inhibitor as ARTs controls, and medium alone (Medium) as negative inhibition control. Culture supernatants were harvested at 24 h post-treatment and the virus loads were determined by measuring cell-free p24. There was no significant change in the virus titer in the culture medium derived from CPP-HuscFv11-treated cells when compared to the cells in medium alone and the other controls (Figure 8A).

The RT activity of the CPP-HuscFv11-exposed virus particles, derived from the treatment assay, was reduced to 48.4% compared to the viruses in medium alone (Medium). The viruses exposed to nucleoside reverse transcriptase inhibitor, Tenofovir, had reduced RT activity to 65.8%, while those of the viruses exposed to protease inhibitor, Darunavir, and control transbodies were not different from those of the viruses in medium alone (Figure 8B).

The virus progeny from infected cells treated with the transbodies and controls were used to infect H9 cells for 4 days and the percent of infected cells (positive for p24) were determined by flow cytometric analysis. The results are shown in Figure 8C. Infectivity of the virus particles obtained from CPP-HuscFv11-treated cells were reduced to 58% compared to the viruses obtained from non-treatment (medium) controls. The infectivity of the viruses exposed to Darunavir and Tenofovir were reduced to 56 and 45%, respectively. The viruses obtained from control CPP-HuscFv-treated cells showed a placebo effect, at11% reduction of virus-producing cells.

## 4. Discussion

HIV-1 progeny are released from infected cells as immature non-infectious particles. The virus maturation process occurs concomitantly with the virus particle release and requires the proteolytic cleavage of Gag and Gag-Pol polyproteins by the virally encoded PR, which is brought into the newly assembled virion as part of the Gag-Pol polyproteins. Maturation requires structural rearrangement within the released virion, including the generation of a condensed conical capsid core [37] and Env clustering on the surface of individual HIV-1 particles [38]. Formation of the conical capsid core within the HIV-1 mature virion requires PR cleavage of Gag and Gag-Pol polyproteins to generate mature structural proteins and viral enzymes. Acquisition of active PR proteolytic activity requires dimerization of Gag-Pol molecules. Dimerization of two Gag-Pol chains in the immature virion contributes to arrangement of two PR domains to form an immature protease dimer [39]. The immature protease dimer autocatalyzes its own N-termini, and releases itself from p6^pol^ transframe domains as an enzyme precursor PR-RT-IN [40,41], followed by enzyme conformational transition into a mature dimer of viral PR [39]. Proteolytic processing at the C-terminus of PR domain embedded in the precursor is mediated by another precursor protease [41]. The active PR cleaves Gag and Gag-Pol polyproteins in the immature particles to yield three major structural proteins, i.e., matrix (MA), capsid (CA), and nucleocapsid (NC), as well as viral enzymes, i.e., RT/RNase H and integrase (IN). Then, there is a dramatic internal structural rearrangement in the virus particle from the immature Gag lattice into the CA lattice [42].

In this study, the human single-chain variable fragments (HuscFvs) that bind to HIV-1 reverse transcriptase connection domain (RTCD) were selected by using phage display technique. The HuscFvs that bound to recombinant RTCD were screened through computerized simulation in order to bin together the HuscFvs that have closely related/similar epitope specificity and to guide selection of the ones that presumptively bound at the RTCD dimer interface and thus could be expected to interrupt the Gag-Pol homodimerization (via RT connection domains), which should consequently hinder HIV-1 progeny maturation [43]. The HuscFvs of the selected *E. coli* clone were then engineered to be cell-penetrable (i.e., become transbodies) by molecular linking to a cell-penetrating peptide (CPP), AA3H [21]. Soluble form of AA3H-HuscFvs was produced in periplasmic space of *E. coli* expression host, such that the transbody molecules acquires natural folding and retained the binding specificity of the non-CPP counterpart. The CPP-linked HuscFvs of the transformed *E. coli* clone 11 (CPP-HuscFv11) at the concentration range used for treatment in this study were biocompatible with the H9 cells which were used as a mammalian cell representative. Besides, the transbodies readily traverse across the usually formidable plasma membrane and co-localized with the intracellular Gag-Pol of the replicating HIV-1.

**Figure 9:**
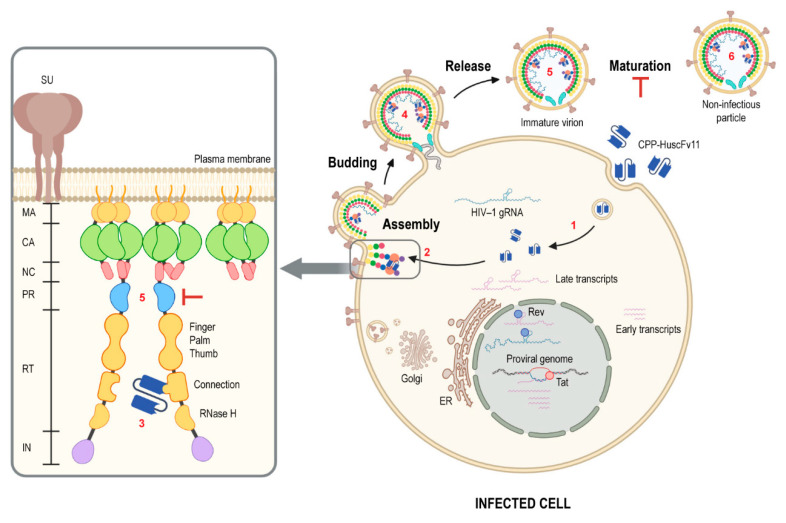
Conceptualized activity of the RTCD-bound CPP-HuscFv11 in the HIV-1 infectious cycle. (**1**) The CPP-HuscFv11 is delivered intracellularly via the endocytic pathway; the transbodies escape into infected cell cytoplasm (CPP function). (**2**) The antibody recognizes its target, which is (**3**) reverse transcriptase connection domain (RTCD) in Gag-Pol polyprotein at the plasma membrane. (**4**) The antibody is trapped in the budded virion through the virus assembly pathway. (**5**) The antibody in the virion prevents virion maturation by interfering with Gag-Pol homodimerization (via RTCD) which hinders virus protease (PR) activation, leaving the immature virus non-infectious (**6**).

Long-term exposure to anti-retroviral therapeutic agents (ARTs) caused markedly adverse effects and worsen serious non-AIDS events, as well as contributed to emergence of drug-resistant/escape mutants. Human antibody fragments offer an alternative approach to ARTs before curative measure for HIV-1 infection could be practically possible. To minimize potential risks of immune reactivity and adverse effects upon long-term use of the transbodies, the human-derived AA3H peptide was used as a carrier for intracellular uptake of the small, “fully human” single-chain antibody molecules (encoded by human V_H_ and V_L_ genes) [20]. The peptide was derived from human membrane-interacting protein, annexin III, which would be safe clinically for repetitive administrations of the CPP-HuscFvs. Additionally, utilizing antibody molecules, which bind multiple residues of the target by using multiple residues in the multiple CDRs, should cope better with the virus mutants than the small chemical inhibitors that tend to interact with the target at a specific site. The CPP-HuscFv11, as well as the ARTs, which were used as anti-HIV-1 controls, including the Darunavir and the Tenofovir, could not reduce the amount of the virus produced from the treated, infected cells. It should be noted that Darunavir at higher concentration (50 nM) could reduce the viral load to 40%, while the Tenofovir could not reduce the viral load at any concentration tested. The CPP-HuscFv11 reduced the reverse transcriptase activity of the virus progeny in, more or less similar magnitude to the Tenofovir drug. The transbodies significantly impaired infectiousness of the newly released HIV-1 progeny in as much as the two ARTs, Darunavir and Tenofovir. Figure 9 illustrates conceptualized activity of the RTCD-bound CPP-HuscFv11. The fully human transbodies should be tested further towards a long-term clinical use as an anti-HIV-1 agent.

## 5. Conclusions

Cell-penetrating fully human single-chain antibodies (transbodies) that bind to HIV-1 reverse transcriptase connection domain (RTCD) in the Gag-Pol polyprotein were produced. The transbodies reduced reverse transcriptase activity as efficiently as the clinically used HIV-1 nucleoside reverse transcriptase inhibitor, Tenofovir. The transbodies reduced the infectiousness of the progeny viruses at a similar magnitude of efficiency as the current ARTs, i.e., the protease inhibitor Darunavir and the Tenofovir. Thus, the fully human transbodies have therapeutic potential for long-term treatment of HIV-1 infection.

## Data Availability

All datasets presented in this study are included in the article/Supplementary Materials.

## Supplementary Materials

The following are available online at https://www.mdpi.com/article/10.3390/vaccines9080893/s1, Figure S1: Circular map for pYidC, Figure S2: Circular map for pET24DAS, Figure S3: Circular map for HIV-1_DA5_ genome organization acquired from de novo assembly, Figure S4: Recombinant RTCD expression cassette and its deduced polypeptide sequence, Figure S5: Direct PCR screening for RTCD-bound phages from *E. coli* clones, Figure S6: Superimposition of models derived from structure minimization methods carried out in this study, Table S1: Specific primers for amplification of overlapped DNA segments of HIV-1_DA5_ genome, Table S2: Model scores of HuscFvs and HIV-1 RTCD obtained from I-TASSER, Table S3: Model scores of HuscFvs and HIV-1 RTCD obtained from ModRefiner. Table S4: Comparison of docking members of the top-ranked clusters that were derived from each docking pair.
