# Supplementary material for: Human Transbodies to Reverse Transcriptase Connection Subdomain of HIV-1 Gag-Pol Polyprotein Reduce Infectiousness of the Virus Progeny
Source: Vaccines (Basel). 2021 Aug 12;9(8):893. doi: 10.3390/vaccines9080893 (PMC8402387; doi:10.3390/vaccines9080893)
Supplement: Supplementary file 1 [file vaccines-09-00893-s001.zip › vaccines-1326936-supplementary.pdf]

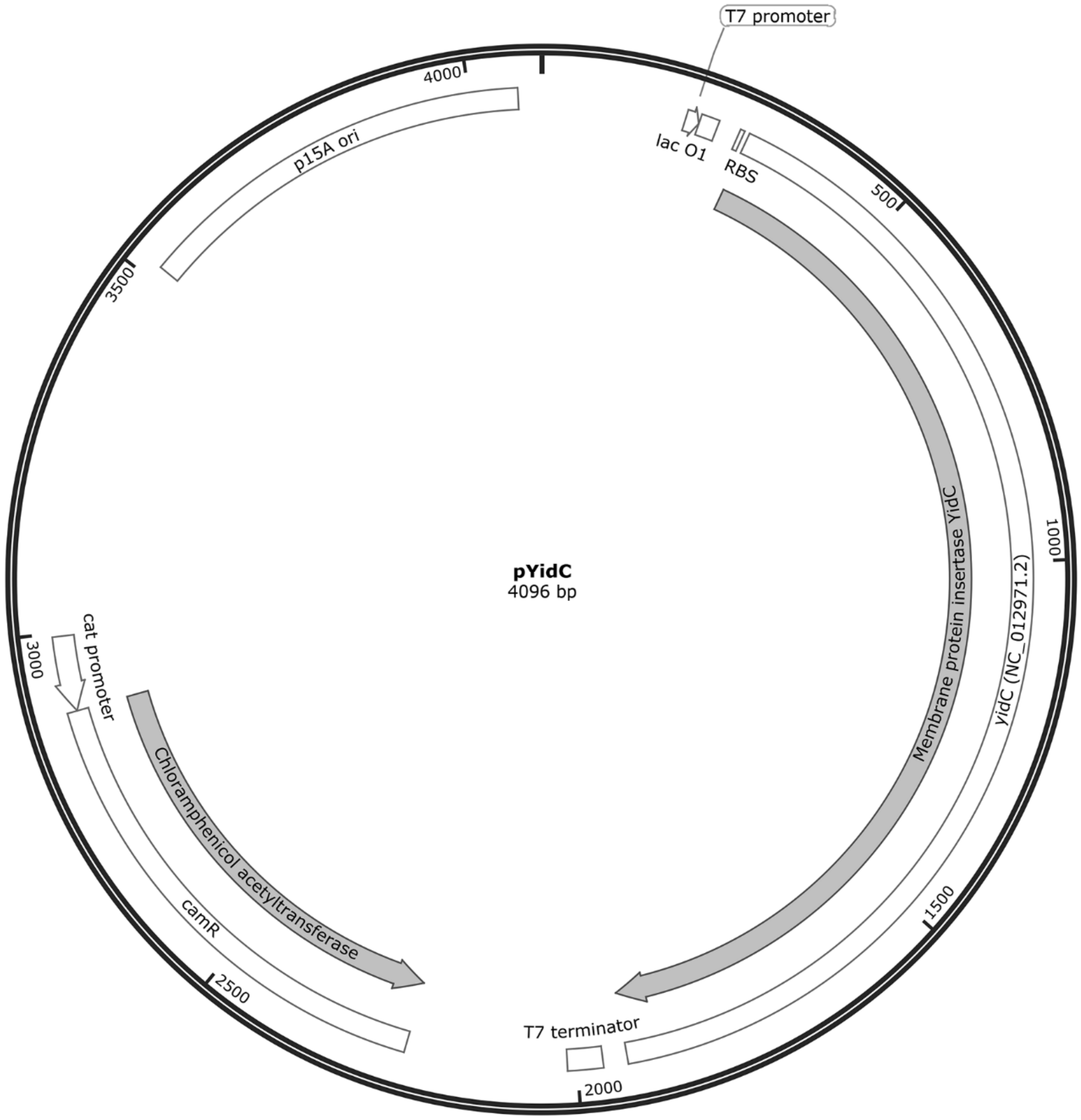

Figure S1. Circular map for pYidC. The vector is a 4096 bp-plasmid carrying *yidC* (accession number NC_012971.2). Gene was cloned into pACYC backbone, which contains chloramphenicol selectable marker (*camR*). The p15A origin allows compatible coexistence with pET vectors and controls the copies number at 20 – 30 copies/cell.

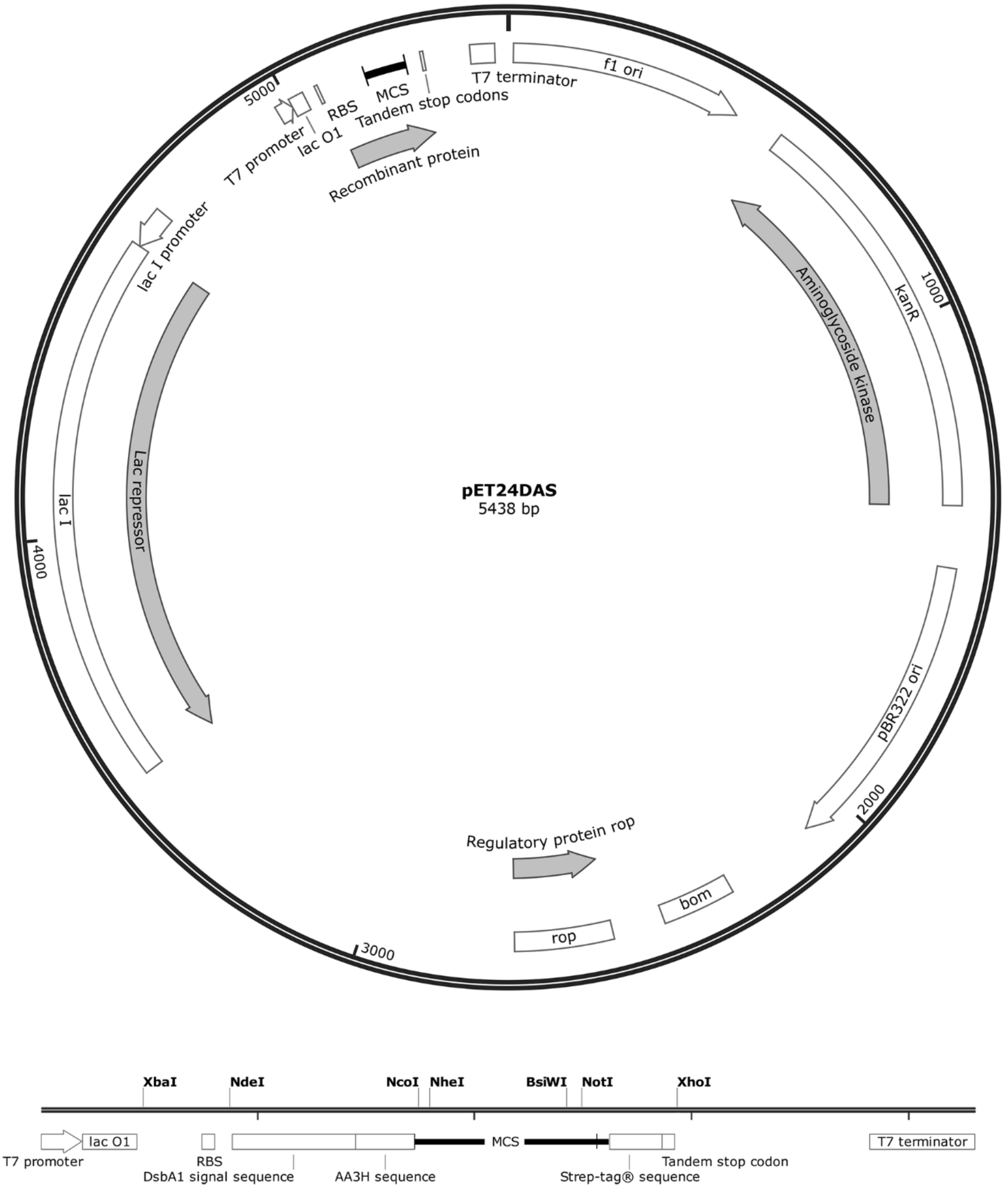

Figure S2. Circular map for pET24DAS. The empty vector is a 5438 bp-plasmid and the map is the same as pET24a(+) with the exception of the sequence between *Nde*I and *Xho*I restriction sites. In this study, the sequence between the *Nde*I and *Xho*I restriction sites was replaced by synthetic DNA encoding DsbA1 signal peptide, AA3H cell-penetrating peptide, multiple cloning site (MCS), Strep-tag II epitope, and tandem stop codons.

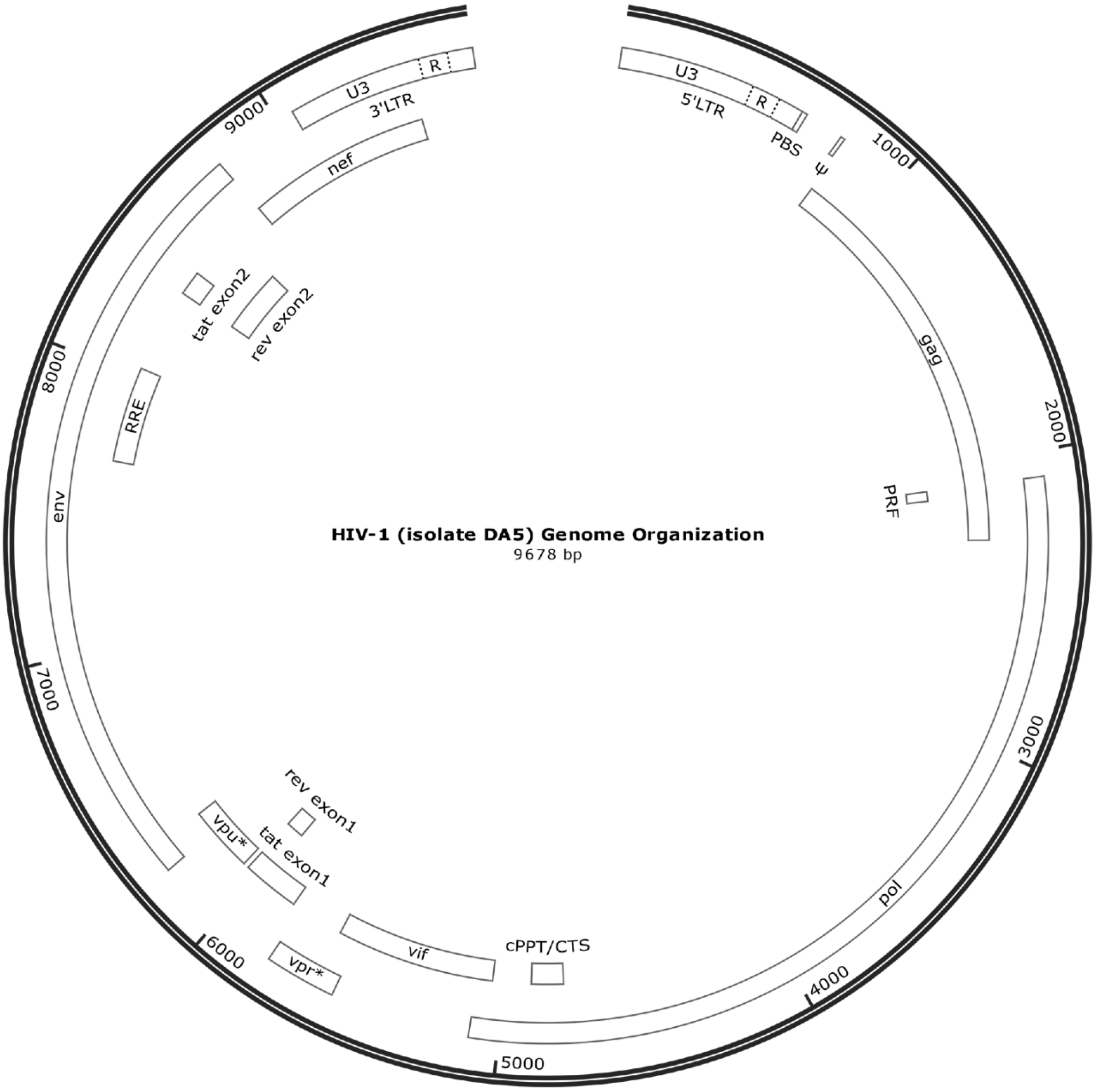

Figure S3. Circular map for HIV-1DA5 genome organization acquired from de novo assembly. LTR, long terminal repeat; PBS, primer binding site; Ψ, Psi packaging element; PRF, programmed−1 ribosomal frameshifting; cPPT, central polypurine tract; CTS, central termination sequence; RRE, Rev response element.

* indicates mutated *vpr*/*vpu*.

A

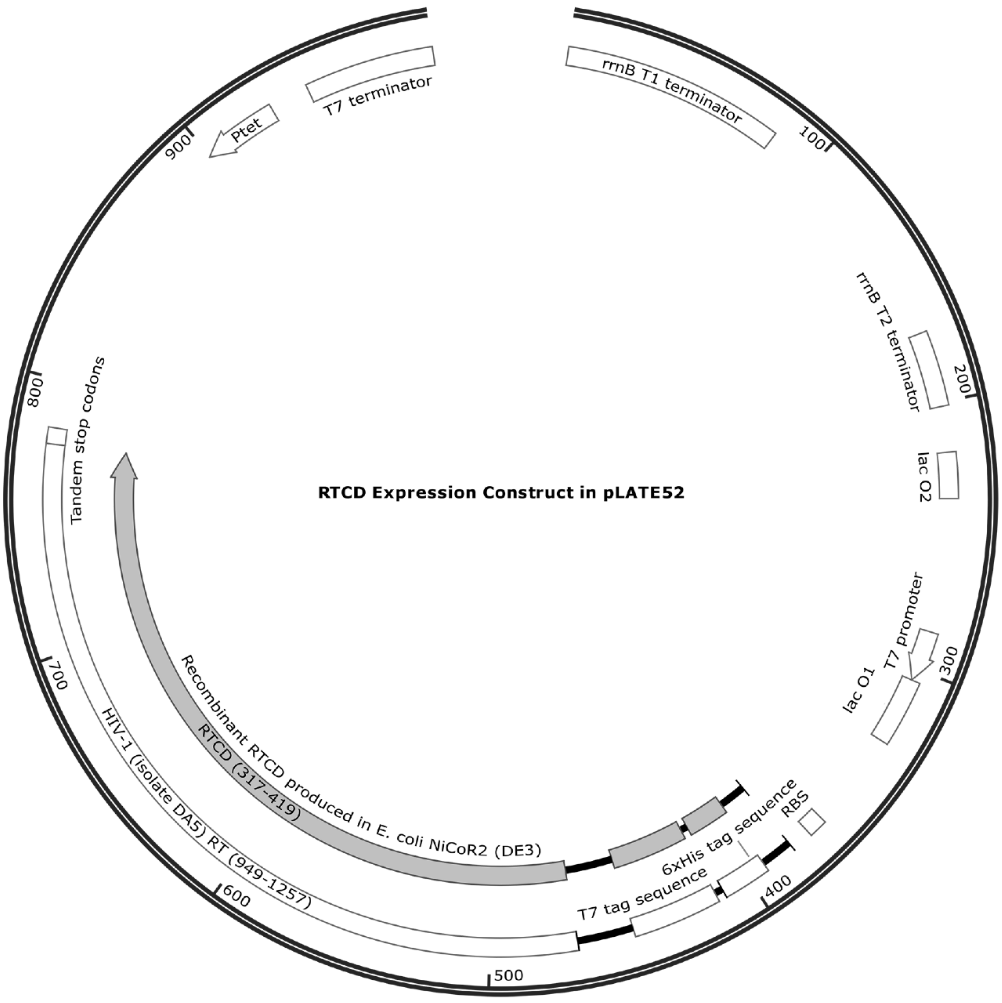

B

```
     6×His  T7 tag            RTCD
MAGSHHHHHHGMASMTGGQQMGRSGWELQVYYDPSKDLVAEVQKQGQDQWTYQIYQEPFKNLKTGK
YARKRSAHTNDVRQLAEVVQKIATESIVIWGKTPKFRLPIQKETWETWWMEYWQATWIPEWEFVNT
```

Figure S4. Recombinant RTCD expression cassette and its deduced polypeptide sequence. (**A**) Schematic diagram of recombinant RTCD expression cassette in pLATE52 vector. Basal level of expression was regulated by *lac* O1 and O2 franked to T7 promoter. Upstream rrnBT1 and T2 terminators prevent a basal gene expression from vector derived promoter-like elements. Downstream of the cloning site is a constitutively induced weak *Tet* promoter (P_{tet}) that operates anti-directionally to the T7 promoter, further reducing the basal expression. (**B**) Deduced polypeptide sequence of recombinant RTCD derived from the pLATE52-RTCD recombinant plasmid. The RTCD was tagged at N-terminus with 6× His and T7 epitope tags.

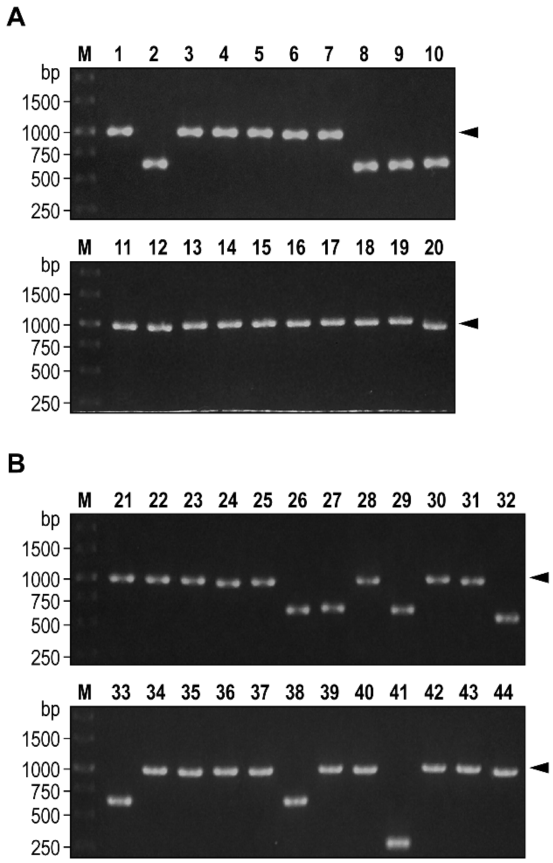

Figure S5. Direct PCR screening for RTCD-bound phages from *E. coli* clones. The phage transformed *E. coli* from two plates (**A** and **B**) that carried *huscfv*-sequences revealed PCR amplicons at about 1 kb (black arrowhead). Lanes M, 1 kb DNA ladder; lanes 1 – 44, phage transformed-*E. coli* clones no. 1-44.

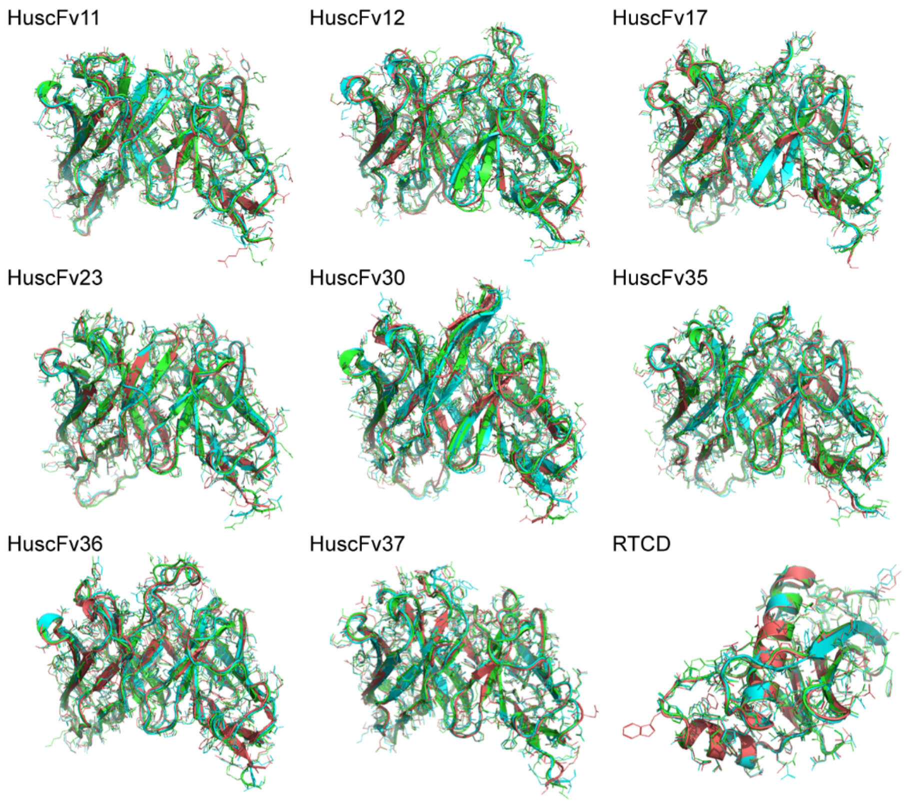

Figure S6. Superimposition of models derived from structure minimization methods carried out in this study. The models obtained from ModRefiner generate slightly deviated protein backbone. The models were further simulated to a native-like state by FG-MD, thus generated variations of side chain-rotameric state of the final models. The figures show structural alignment of model F1 (red), model F2 (green), and model F3 (cyan) from the selected HuscFvs and RTCD. All models were claimed to be energy minimized-near native state homology models.

Table S1. Specific primers for amplification of overlapped DNA segments of HIV-1$_{DA5}$ genome.

Target	Primer Name	Sequence (5′–3′)
RU5-PR	RU5-PR-forward	GGTCTCTCTTGTTAGACCAGG
	RU5-PR-reverse	GAAATTTAAAGTACAACCAATCTGAGTC
PR-IN	PR-IN-forward	CCTCAAATCACTCTTTGGCAACGAC
	PR-IN-reverse	CTAATCCTCATCCTGTCTACCTGCCAC
IN-NT	IN-NT-forward	CAATGTCCAACAGGAATTTGGG
	IN-NT-reverse	CTGCTTTGGTATAGGATTTTGATGATC
NT-CT	NT-CT-forward	ATGGAGCTGGTAGATCCTAACCTAGAG
	NT-CT-reverse	CTAAGCGCATGGATCTGTCTCTGC
CT-RU3	CT-RU3-forward	CCATCATCAGAGGGAACCCGAC
	CT-RU3-reverse	CTCAAGGCAAGCTTTATTGAGGCTTTAAGC

Table S2. Model scores of HuscFvs and HIV-1 RTCD obtained from I-TASSER.

Model	C-score	TM-score	RMSD	No. of decoys	Cluster density
HuscFv11	0.96	0.84 ± 0.08	3.8 ± 2.6	9978	0.6655
HuscFv12	0.89	0.83 ± 0.08	4.0 ± 2.7	10147	0.5652
HuscFv17	1.29	0.89 ± 0.07	3.2 ± 2.3	10125	0.8943
HuscFv23	1.29	0.89 ± 0.07	3.2 ± 2.3	10177	0.9154
HuscFv30	1.22	0.88 ± 0.07	3.4 ± 2.4	10178	0.8113
HuscFv35	1.30	0.89 ± 0.07	3.2 ± 2.3	10193	0.8766
HuscFv36	1.35	0.90 ± 0.06	3.1 ± 2.2	10190	0.8841
HuscFv37	1.27	0.89 ± 0.07	3.3 ± 2.3	10160	0.8156
RTCD	1.52	0.93 ± 0.06	1.2 ± 1.2	10200	1.0000

Table S3. Model scores of HuscFvs and HIV-1 RTCD obtained from ModRefiner.

Model	RMSD			TM-score		
	**M1**	**M2**	**M3**	**M1**	**M2**	**M3**
HuscFv11	0.620	0.660	0.670	0.9906	0.9897	0.9891
HuscFv12	0.542	0.709	0.690	0.9918	0.9877	0.9868
HuscFv17	0.551	0.541	0.608	0.9917	0.9924	0.9916
HuscFv23	0.740	0.636	0.696	0.9891	0.9916	0.9912
HuscFv30	1.237	0.801	0.809	0.9670	0.9827	0.9858
HuscFv35	0.591	0.566	0.517	0.9903	0.96916	0.9923
HuscFv36	1.026	0.712	0.563	0.9819	0.9866	0.9914
HuscFv37	0.651	0.424	0.748	0.9905	0.9949	0.9860
RTCD	0.267	0.242	0.339	0.9950	0.9958	0.9933

Table S4. Comparison of docking members of the top-ranked clusters that were derived from each docking pairs.

HuscFv	Docking pair*								
	**1 – 1**	**1 – 2**	**1 – 3**	**2 – 1**	**2 – 2**	**2 – 3**	**3 – 1**	**3 – 2**	**3 – 3**
11	82**	131	128	104	126	112	125	131	**136**
12	**365**	105	170	275	111	218	122	145	117
17	113	**184**	94	108	109	138	139	129	161
23	132	81	72	145	107	116	**145**	114	101
30	168	83	87	**217**	168	102	197	153	91
35	143	95	100	**158**	122	**158**	155	115	86
36	120	121	209	123	169	180	**306**	143	196
37	156	94	138	87	112	122	**258**	197	196

* 1 – 1, docking between HuscFv model F1 and RTCD model F1; 1 – 2, docking between HuscFv model F1 and RTCD model F2; and so on.

** Number of the docking member, which was obtained from the top-ranked docking cluster of ClusPro 2.0. The green numbers indicate the highest docking members in the clusters compared to other docking pairs.
